# Symmetry Breaking of Counter-Propagating Light in a Nonlinear Resonator

**DOI:** 10.1038/srep43142

**Published:** 2017-02-21

**Authors:** Leonardo Del Bino, Jonathan M. Silver, Sarah L. Stebbings, Pascal Del'Haye

**Affiliations:** 1 National Physical Laboratory (NPL), Teddington, TW11 0LW, United Kingdom

## Abstract

Spontaneous symmetry breaking is a concept of fundamental importance in many areas of physics, underpinning such diverse phenomena as ferromagnetism, superconductivity, superfluidity and the Higgs mechanism. Here we demonstrate nonreciprocity and spontaneous symmetry breaking between counter-propagating light in dielectric microresonators. The symmetry breaking corresponds to a resonance frequency splitting that allows only one of two counter-propagating (but otherwise identical) states of light to circulate in the resonator. Equivalently, this effect can be seen as the collapse of standing waves and transition to travelling waves within the resonator. We present theoretical calculations to show that the symmetry breaking is induced by Kerr-nonlinearity-mediated interaction between the counter-propagating light. Our findings pave the way for a variety of applications including optically controllable circulators and isolators, all-optical switching, nonlinear-enhanced rotation sensing, optical flip-flops for photonic memories as well as exceptionally sensitive power and refractive index sensors.

Bi-directional propagation of light is an important prerequisite for many types of optical elements including interferometers and resonators. At the same time, nonlinear optical effects have driven many photonic developments in the past decades. Interaction between counterpropagating light has been observed for example in nonlinear loop mirrors^1^,^2^ and through gain competition in ring lasers^3^,^4^,^5^. In this work we demonstrate direct Kerr-nonlinearity-mediated light-with-light interaction between counterpropagating modes in a passive ultra-high-Q microresonator. This leads to a spontaneous symmetry breaking between the resonator’s clockwise and counterclockwise eigenmodes. In our experiments, a whispering gallery resonator provides the power enhancement to achieve the required light intensities. Such an effect has been theoretically predicted in the 1980 s by Kaplan and Meystre in the context of nonlinear enhanced Sagnac interferometry for rotation sensing^6^. Theoretical calculations show that the nonlinear interaction could enable significantly enhanced rotation sensors^7^,^8^ that are limited only by the relative power stability between the counter-propagating light. In addition the symmetry breaking could be used for precise optical power and refractive index sensing^9^. Equally important, the interaction between counter-propagating waves leads to controllable non-reciprocal propagation of light. In particular, integrated photonic circuits require new ways of achieving non-reciprocal light propagation that do not rely on magneto-optical effects^10^. This has been demonstrated using metallic-silicon waveguides^11^, asymmetric backscattering in microresonators^12^, coupled PT-symmetric resonator systems^13^,^14^, in asymmetrically coupled systems of microrings^15^, and a variety of other devices^16^,^17^,^18^,^19^. Our results rely on direct interaction between counterpropagating light in a Kerr resonator and enable the development of novel types of integrated optical elements including optically controllable diodes, circulators and all-optical flip-flops^20^,^21^,^22^,^23^. In our measurements we show a detailed analysis of the symmetry breaking, which manifests itself as a resonance splitting between the clockwise and counterclockwise microresonator modes. In addition, we perform a threshold power analysis of the nonlinear interaction between counter-propagating light and demonstrate all-optical switching between clockwise and counterclockwise circulating light. Our experimental findings are in excellent agreement with theoretical calculations of Kerr-nonlinearity-induced symmetry breaking and non-reciprocal propagation of light.

The nonlinear interaction between counter-propagating light in a nonlinear Kerr medium is illustrated in Fig. 1a. Crucially, two counter-propagating light waves with equal wavelength outside of a nonlinear Kerr medium will have different wavelengths within the medium if their powers are unequal. This can be explained by cross-phase modulation between the two light waves, in which the weaker light wave experiences a stronger refractive index change. More specifically, the refractive index changes ∆*n*_A, B_ induced by the nonlinear interaction are given by


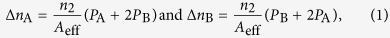


with subscripts A, B indicating the two counter-propagating waves with powers *P*_A, B_, *n*_2_ being the nonlinear refractive index of the medium and *A*_eff_ being the effective mode cross-section. It is important to note that a counter-propagating light wave induces twice the refractive index change compared to the change induced by self-phase-modulation^24^,^25^. In the case of an optical resonator with *x*^(3)^ nonlinearity, the difference in refractive index change leads to two different optical path lengths experienced by the counter-propagating modes. This is reflected in a splitting of the resonance frequencies of the clockwise (CW) and counterclockwise (CCW) modes as shown in Fig. 1c,d. Theoretical predictions suggest that such resonators exhibit symmetry breaking when simultaneously pumped with equal power in both the CW and CCW directions^6^,^7^. This may be explained by a self-amplification of small power fluctuations between the counter-propagating light. With the laser frequency at higher frequency compared to the resonance, the optical mode with infinitesimally lower power experiences a stronger Kerr-shift and is pushed further away from the pump laser frequency. Simultaneously, the stronger mode experiences less Kerr-shift and moves towards the pump laser, such that it gains even more power. This increases the resonance splitting until the system comes to a new equilibrium and self-phase modulation prevents the stronger mode from further approaching the laser (similar to thermal self-locking^26^ of microresonator modes to a laser). For low optical powers (Fig. 1b), no splitting is induced and the CW and CCW circulating powers remain equal. Above a certain threshold power however, the state with equal coupled powers becomes unstable, and the system instead chooses one of the two states shown in Fig. 1c and d, breaking the CW-CCW symmetry. Note that the induced mode splitting should not be confused with mode splitting through backscattering of light^27^,^28^. Backscattering of light in the resonator is a symmetric process such that each of the split resonances contains a superposition of the clockwise and counterclockwise propagating modes.

The optical powers that are coupled into the clockwise and counterclockwise circulating modes of a whispering gallery resonator are given by the following coupled equations (see Supplementary information for further details):


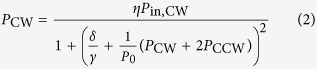



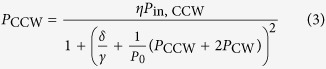


Here, *P*_in, CW_ and *P*_in, CCW_ are the incident pump powers, *δ* is the detuning of the laser frequency with respect to the resonance without Kerr shift, *γ* = *γ*_0_ + *κ* is the loaded half-linewidth of the resonance for intrinsic and coupling-related decay rates *γ*_0_ and *κ*, and *η* = 4*κγ*_*0*_/*γ*^2^ is the coupling efficiency. The quantity *P*_0_ = *πn*_*0*_*A*_eff_/(*QF*_0_*n*_2_) is the characteristic coupled power at which Kerr nonlinear effects occur, with *n*_0_ and *n*_2_ being the linear and nonlinear refractive indices for a resonator with effective cross-sectional area *A*_eff_, loaded quality factor *Q*, and intrinsic finesse *F*_0_. Importantly, equations (2 show that the nonlinear interaction with the counter-propagating light wave is twice as strong as the self-phase-modulation-induced interaction. This can be seen from the factor of two in the term *P*_CW_ + 2*P*_CCW_ in equation (2). Moreover, an analysis of equations (2 shows that for 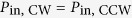, symmetry breaking occurs when 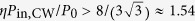 over a range of laser detunings *δ*/*γ* that depends on the value of 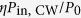. The threshold incident power for symmetry breaking is thus:


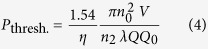


where *V*≈2*πRA*_eff_ is the mode volume for resonator radius *R, λ* is the vacuum wavelength of the laser and *Q*_0_ is the intrinsic quality factor.

Figure 2a shows our experimental setup, which is based on a 2.7 mm diameter high-Q whispering-gallery-mode fused silica microrod resonator^29^. The resonator has an intrinsic quality factor *Q*_0_ of approximately 7 × 10^7^ and an effective cross-sectional area *A*_eff_ of approximately 60 μm^2^ (see Supplementary information). Light from an amplified continuous wave external cavity diode laser (ECDL) in the 1.55 μm wavelength range is coupled into the resonator in both directions via a tapered optical fibre^30^. A fibre-coupled electro-optic intensity modulator allows us to modulate the ratio of the pump powers *P*_in, CW_ and *P*_in, CCW_.

The spontaneous symmetry breaking is demonstrated in Fig. 2b. Sweeping the laser frequency across the resonance with *P*_in, CW_ = *P*_in, CCW_, the coupled powers *P*_CW_ and *P*_CCW_ initially follow the same trace before abruptly diverging, randomly choosing one of two states. Note that the total resonance shift of ~300 MHz also includes a thermally induced component^26^,^31^ and a unidirectional Kerr shift^32^, which does not contribute to a resonance splitting. We observe threshold powers of ~10 mW, though this could be easily reduced to tens of microwatts by using chip-based resonators. In state of the art silicon nitride resonators^33^,^34^ the threshold power could be as low as 50 μW (assuming 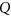 = 1.7 × 10^7^, *n*_2_ =  2.4 × 10^–15^ cm^2^/W, diameter = 50 μm, *A*_eff_ = 1 μm^2^). An interesting feature of the nonlinearity induced symmetry breaking is hysteresis, which is shown in Fig. 2c. Holding the laser frequency constant within the bistable regime, we modulate *P*_in, CCW_ at 5 kHz by ± 25%. The observed hysteresis can be explained by the resonance frequency splitting in the two stable states. To overcome the resonance splitting and switch into the other state, the initially weaker pump direction has to be significantly increased to a power well above that of the counter-propagating pump light. Notably, this hysteresis allows the system to be used as an all-optical flip-flop or binary memory unit^20^,^21^. Since this process is mediated by the almost instantaneous Kerr effect, the switching times are only limited by the cavity lifetime. In the presented data this is on the order of 30 ns, however this could be significantly reduced in a material with lower Q-factor and higher nonlinearity (e.g. < 1 ns in a silicon nitride resonator with Q = 3 × 10^6^, diameter = 50 μm, *A*_eff_ = 1 μm^2^ at 2 mW optical power). Moreover, the non-reciprocal light propagation in each of the stable states can be exploited to realise optically switchable circulators or isolators (cf. Fig. 2b).

The amplification of power imbalances is tested against the theory in Fig. 3a and b. Here the laser frequency is scanned across the resonance for a range of total pump powers, while keeping the ratio *P*_in, CCW_/*P*_in, CW_ fixed at 0.9. At low pump powers, no resonance frequency splitting is observed and the two counter-propagating modes show the same profiles when sweeping across the resonance (lowest power curve in Fig. 3a). Increasing the power, the effect of the cross-phase modulation induced Kerr shift leads to a large difference between the coupled powers. The theoretical results shown in Fig. 3b use the same parameters as employed experimentally in Fig. 3a. Included in the calculations is a thermally induced frequency shift^26^,^31^. A fit of the maximum coupled power difference vs. total pump power (Fig. 3c) is in excellent agreement with the measured data. Small deviations between the curves in Fig. 3a and b may be attributed to modelling the system using the assumption that the thermal shift of the resonance frequency is immediate, whereas in reality it has a delayed response^31^.

Figure 4 shows additional comparisons between our experimental results and theoretical simulations of the symmetry breaking between counterpropagating light in resonators. Figure 4a includes colour-coded solutions for launching unequal powers into a microresonator.

In conclusion, we demonstrate the observation of spontaneous symmetry breaking between counter-propagating light in a nonlinear resonator by pumping an optical microresonator equally in the clockwise and counterclockwise directions. Our results closely match our theoretical predictions based on nonlinear interaction between counter-propagating light induced by the Kerr nonlinearity. We show that above a threshold power, a symmetry-broken regime exists as first predicted by Kaplan and Meystre^6^,^7^. Several potentially far-reaching applications are proposed for this effect. The observed symmetry breaking represents arguably the most fundamental way to induce optical non-reciprocity, which is urgently needed for chip-integrable optical diodes and circulators for forthcoming waveguide coupled lasers. Moreover, the observed hysteresis will allow the realisation of all-optical flip-flops^20^,^21^ for photonic memories and data processing. Calculations show that smaller resonators with higher nonlinearity (e.g. state of the art silicon nitride resonators^34^) enable threshold powers of a few tens of microwatts and switching speeds exceeding 1 GHz at power levels of a few milliwatts. In addition, just below the threshold of the symmetry breaking, the state of the system is exceptionally sensitive to minute imbalances between the clockwise and counter-clockwise circulating light^6^. This enables a range of enhanced sensors for optical power, refractive index^9^ and rotation^6^,^8^, as well as new types of near-field probes. Besides these immediate practical applications, the symmetry breaking shows significant potential for a variety of novel concepts in basic research and for future advanced integrated photonic circuits. Most importantly, the fundamental nature of symmetry breaking in resonant states with periodic boundary conditions through nonlinear coupling could impact science beyond optical physics e.g. in acoustic waves, quantum mechanical wave functions, and microwave resonators.

Note added: we would like to draw the reader’s attention to a related recent work^35^.

## Additional Information

**How to cite this article:** Del Bino, L. *et al*. Symmetry Breaking of Counter-Propagating Light in a Nonlinear Resonator. *Sci. Rep.* **7**, 43142; doi: 10.1038/srep43142 (2017).

**Publisher's note:** Springer Nature remains neutral with regard to jurisdictional claims in published maps and institutional affiliations.

## Supplementary Material

Supplementary Information

## Figures and Tables

**Figure 1:**
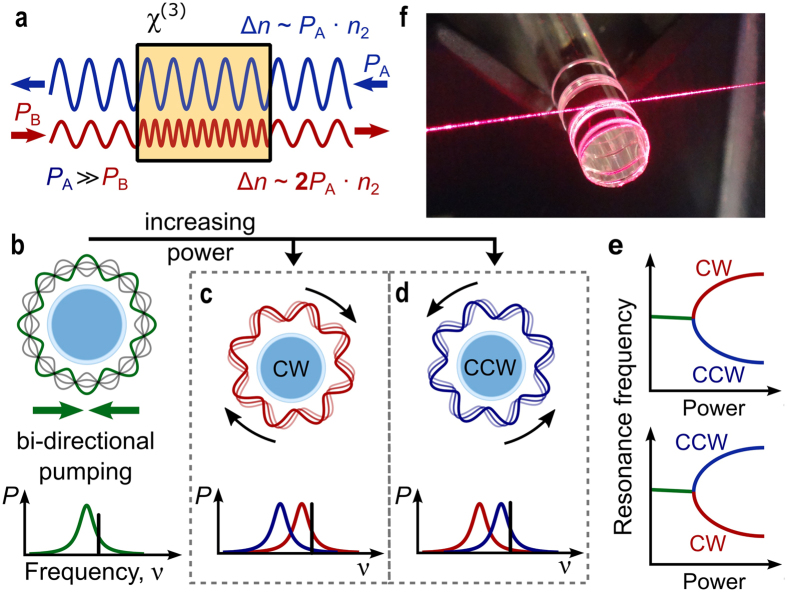
Nonlinear interaction between counter-propagating light. (**a**) Principle of Kerr-nonlinearity-mediated interaction between counter-propagating light without a resonator. Two counter-propagating but spatially overlapping light waves (offset in the diagram for clarity) with identical frequency will experience a different effective refractive index change ∆*n* depending on their powers (*P*_A_, *P*_B_). The light wave with lower optical power (*P*_B_) experiences a stronger refractive index increase, which leads to a shorter wavelength. (**b**) Bi-directional pumping of a whispering gallery resonator at low power, generating a standing wave. When increasing the power, the system collapses either into state (**c**) with clockwise propagating (CW) light or state (**d**) with counterclockwise (CCW) propagating light. This symmetry breaking goes along with a resonance frequency splitting between the counter-propagating optical modes (shown in the lower part of panels (**b**–**d**) where the black line denotes the pump frequency). (**e**) Symmetry breaking shown by the splitting between the CW and CCW resonance frequencies with increasing power. (**f**) Fused silica resonator and tapered fibre used in the experiments (highlighted with red laser light).

**Figure 2:**
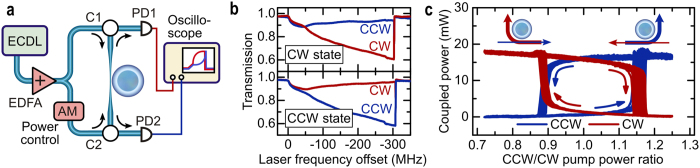
Experimental setup and data showing nonlinearity-induced symmetry breaking between counter-propagating light. (**a**) Schematic of the setup. The light of an external cavity diode laser (ECDL) is split into two parts and sent into a fused silica whispering gallery resonator from opposite directions. Two optical circulators C1, 2 split off the light coming from the microresonator and enable the measurement of the clockwise and counterclockwise transmission using photodiodes PD1 and PD2. The ratio between the counter-propagating pump powers can be adjusted using an amplitude modulator (AM). (**b**) Transmission vs. laser frequency measured on two consecutive laser sweeps across the resonance with equal powers of 80 mW launched in each counter-propagating direction. The system spontaneously breaks the symmetry and “picks” one of two possible states allowing light to couple in from just one direction, i.e. CW (top) or CCW (bottom). (**c**) Measurement of optical switching between the CW and CCW states, showing a hysteresis loop. The graph is obtained by tuning the laser into resonance and modulating the power in one direction up and down ~30 times. Inset: illustrations of the power flow in the two states.

**Figure 3:**
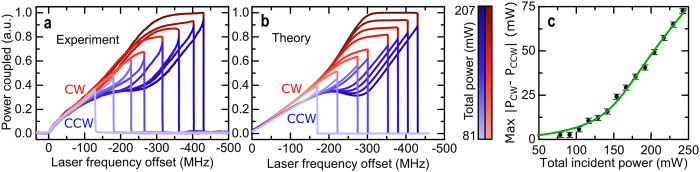
Threshold and power dependence of the symmetry breaking between clockwise and counterclockwise propagating light. (**a**) Amount of light coupled into the resonator as a function of the laser frequency for increasing incident powers (by changing the EDFA output power, cf. Fig. 2). Higher powers (shown as darker colours) lead to an increased splitting between the clockwise (red) and counterclockwise (blue) modes. The incident power is 10% higher in the clockwise direction to avoid switching caused by fluctuations in the relative power. (**b**) Theoretical calculation of the power coupled into the resonator with the same parameters as in panel (**a**). (**c**) Maximum difference between clockwise and counterclockwise coupled powers as a function of the total input power. The black dots show the experimental measurements while the green curve is a theoretical fit for a pump power ratio of 0.9.

**Figure 4:**
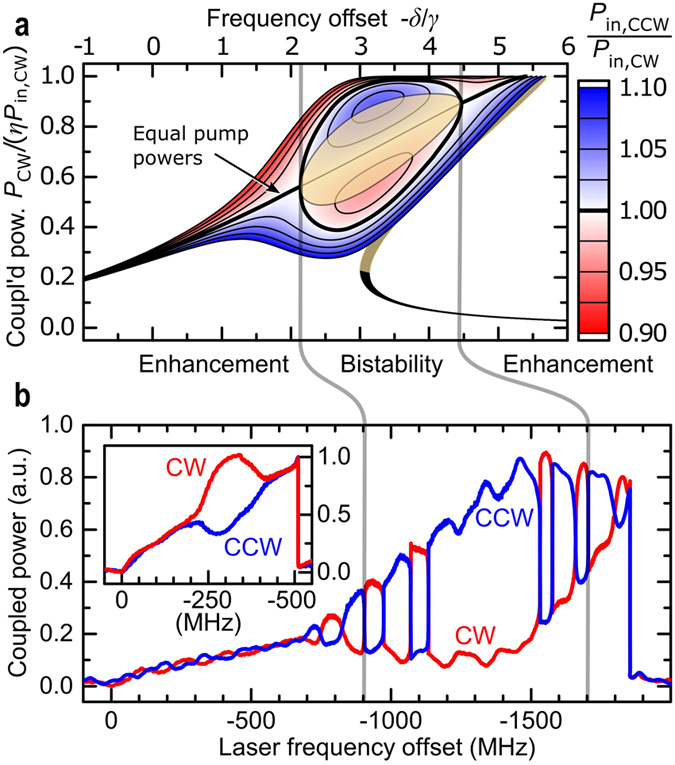
Different regimes of nonlinearity-induced mode splitting. (**a**) Theoretical prediction for the power *P*_CW_ coupled into the resonator in the clockwise direction as a function of the normalised laser detuning frequency *δ*/*γ*. The incident power imbalance is colour-coded with red (blue) contours corresponding to higher power in the CW (CCW) direction. The bold black curve shows spontaneous symmetry breaking in the case of equal pump powers. In this region between the two grey lines, two stable solutions exist for a range of power imbalances. The beige shaded area contains unstable solutions. (**b**) Scan of the laser frequency across the resonance while backscattering-induced interference effects in the setup cause *P*_in, CCW_/*P*_in, CW_ to change about unity by ±3% at a period of ~170 MHz in the laser frequency. The launched power is 180 mW in each direction. Outside the grey lines the coupled power difference is enhanced with respect to the incident power imbalance by the non-reciprocal Kerr shift. The enhancement increases approaching the bistable regime, in which the system begins to jump between the two stable configurations. The inset shows a sweep across a microresonator mode with equal power and no interference effects, leading to a “bubble”-shaped symmetry-broken region corresponding to the bold black trace in panel (a).
