# Supplementary material for: Symmetry Breaking of Counter-Propagating Light in a Nonlinear Resonator
Source: Sci Rep. 2017 Feb 21;7:43142. doi: 10.1038/srep43142 (PMC5318886; doi:10.1038/srep43142)
Supplement: Supplementary Information [file srep43142-s1.pdf]

Supplementary Information for

Symmetry Breaking of Counter-Propagating Light in a Nonlinear Resonator

L. Del Bino, J. M. Silver, S.L. Stebbings, P. Del'Haye

correspondence to: pascal.delhaye@npl.co.uk

This PDF file includes:

S1. Supplementary Methods

S2. Supplementary Text

Fig. S1 to S4

1. Supplementary Methods

Fabrication of microresonators

Our microresonator is produced by milling a 2.7 mm diameter fused silica rod with a commercial CO_2 laser (25 W) to create a rotationally-symmetric protrusion around the rod[1]. The rod is fixed on a spindle and the laser is focused onto its surface, steered using two galvo mirrors to obtain the desired microresonator profile. The machining is divided into three steps: first we use high power to evaporate the glass and make a cylindrical surface coaxial with the spindle, then we reduce the power and start alternating the position of the laser between two different focal spots 120 µm apart until two grooves appear in the rod and the central prominence is well defined (see Fig. S1). Finally, we reduce the laser power to melt the glass surface instead of evaporating it, in order to obtain the highest possible smoothness and thus a high Q factor. This fabrication process produces an almost perfect rotational symmetry that nearly cancels any kind of backscattering due to imperfections. This can be seen from the absence of mode splitting when sweeping the laser frequency across any resonance at low power. In addition, we don't measure a significant amount of backscattering (less than -10 dB) when we send light in the resonator just in one direction.

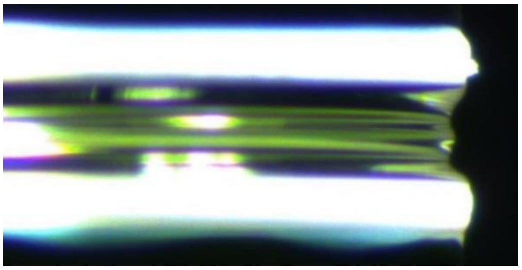

Fig. S1 | Microrod resonator used in the experiment. The resonator is machined from a 2.7 mm diameter fused silica rod by evaporating and melting its surface with a CO_2 laser while spinning it on a motorised spindle.

Fabrication of tapered fibres

The tapered fibre in our experiment is obtained from a commercial 125 µm diameter optical fibre. A 3 cm section of the fibre, stripped of the plastic coating, is heated up to the softening point of glass and stretched by 6 cm using two linear motors. By optimising the temperature and pulling speed, we obtain tapered fibres that allow over-coupling to the resonators. The diameter of the tapered fibre is thus estimated to be of the order of, or less than, 1 µm. After tensioning, the tapered fibre is glued to an aluminium bracket that will be fixed to the experiment setup, and FC-APC connectors are spliced onto each end. The

resonator is fixed to a 3-axis micrometer translation stage that allows us to move it with respect to the fixed tapered fibre to tune the coupling strength.

Experimental setup

We send light into the resonator in opposite directions by splitting a fibre-coupled and amplified mode-hop-free diode laser in the 1.55 μm wavelength range into two paths. To be able to detect the transmission we use circulators at both ends of the tapered fibre to separate out the transmitted light in each direction. We also employ 1% couplers to measure the incident power in each direction. A variable attenuator and a fibre-coupled electro-optic intensity modulator control power in the two arms, allowing us to tune and modulate the pump power imbalance. A fibre-loop interferometer fed with light from the laser provides the calibration for the frequency offset (see “Calibrating the laser frequency axis” below).

Measuring the Q factor, finesse and effective mode cross-sectional area

It is possible to determine the intrinsic and loaded Q factors and finesses of a resonance via its linewidth and coupling efficiency (in the limit of high Q factors). This is done by modulating sidebands onto the laser using an electro-optic modulator (EOM), sweeping the frequency across the resonance and fitting the sum of three Lorentzians to the transmission profile (see the red curve in Fig. S2). By measuring the distance between the sidebands and scaling this to twice the modulation frequency, we are able to calibrate the linewidth of the carrier. This measurement is performed at very low power in order to avoid Kerr and thermal broadening. To ensure that this is the case, we take measurements while scanning the laser frequency in both directions across the resonance and verify the absence of thermal or Kerr-induced resonance broadening when scanning from high to low laser frequencies. Knowing the laser frequency and the mode family’s free spectral range (FSR) we find the loaded Q factor and finesse F, and using the coupling efficiency η obtain their intrinsic counterparts as

$$Q_0 = \frac{2Q}{1 \pm \sqrt{1-\eta}} \qquad\qquad F_0 = \frac{2F}{1 \pm \sqrt{1-\eta}} \tag{S1}$$

where the $+$ sign is for an undercoupled resonator and the $-$ sign is for an overcoupled one.

The Kerr broadening is linked to the Kerr effect that shifts the resonance to lower frequencies with increasing circulating power by increasing the refractive index. If we

sweep the laser frequency from above to below the resonance at a sufficiently high speed to avoid thermal broadening, but slower than the cavity build-up time, the power P transmitted by the tapered fibre will have the shape of a tilted Lorentzian satisfying:

$$P = P_0 - \frac{A}{1 + \frac{4}{\Delta\nu^2}(\nu - \nu_0 - KP)} \tag{S2}$$

where $\Delta\nu$ is the full linewidth in terms of frequency ν, A is the height of the peak and K is the degree of tilt. By fitting this curve, it is possible to derive the effective mode cross-sectional area A_{eff} from K as explained below.

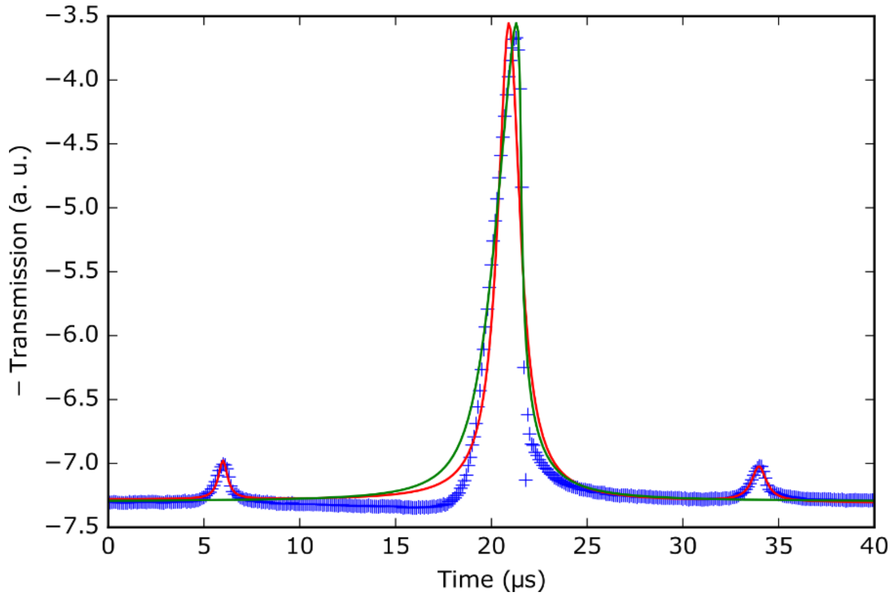

Fig. S2 | Transmission of the tapered fibre in one direction while sweeping the laser frequency across a resonance with 40 MHz sidebands modulated. The sum of three Lorentzians (red) is fitted to calibrate the *x*-axis via the separation of the sidebands, and then a tilted Lorentzian (green, Eqn. S2) is fitted to the central region in order to measure the linewidth and Kerr shift, from which the effective mode cross-sectional area is derived.

An example of such a measurement is shown (with the transmission axis inverted) in Fig. S2. We modulate 40 MHz sidebands onto the laser while sweeping the carrier frequency from above to below the resonance and recording the transmission of the tapered fibre. The horizontal axis is calibrated in terms of frequency by fitting the sum of three Lorentzians as above, and the vertical axis is expressed as optical power via the calibration of the photodiode used to measure it. The coupling efficiency η is found by dividing the

sum of the heights of all three peaks by the transmission far away from the resonance. A tilted Lorentzian (Eqn. S2) is then fitted to the central peak. From the linewidth $\Delta\nu$, the coupling efficiency η and the laser frequency ν_{laser} we obtain the intrinsic finesse F_0. The effective mode cross-sectional area is then given by

$$A_{\text{eff}} = \frac{F_0 n_2 \nu_{\text{laser}}}{2\pi n_0 K} \quad \text{(S3)}$$

where n_0 and n_2 are the linear and nonlinear refractive indices of the resonator respectively.

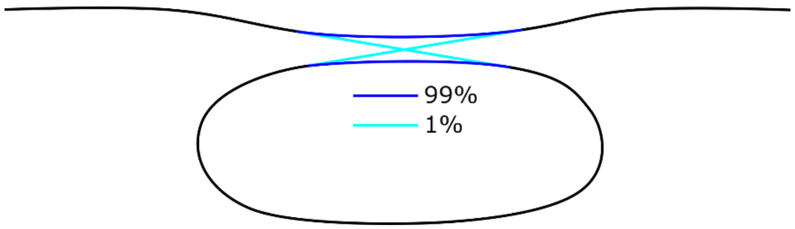

Fig. S3 | Fibre loop resonator. The frequency of the external-cavity diode laser (ECDL) is calibrated using a fibre loop resonator formed by connecting a 1% fibre coupler to itself.

Calibrating the laser frequency axis

The data for Fig. 2b, 3a-b and 4b are taken by modulating the current of the external-cavity diode laser (ECDL) to sweep its frequency. During this modulation, the optical power is kept constant by subsequently sending the light through an erbium doped fibre amplifier, which is operated in saturation. The ECDL laser current modulation is produced by feeding a triangular wave voltage from a signal generator to the laser controller. By recording this voltage at the same time as the transmission of the tapered fibre, we are able to use it to associate a frequency offset with each data point.

However, the laser frequency does not vary linearly with the ECDL current. We therefore perform a further step in order to extract the frequency offset. To get an equally spaced frequency reference we send a small amount of the pump light through a fibre loop cavity consisting of 1% coupler connected to itself as shown in Fig. S3. The mode spacing of the fibre loop interferometer was itself calibrated against the sidebands generated at known frequencies using the EOM. Using the fibre loop cavity as a reference we are able to produce a calibration curve between the modulation voltage and the laser frequency

offset, which we apply to the microresonator data to generate the laser frequency axis.

S2. Supplementary Text

Symmetry breaking

Under the condition of equal pump powers $P_{\mathrm{in,CW}} = P_{\mathrm{in,CCW}} \equiv P_{\mathrm{in}}$, the solutions to the coupled equations (1, 2) are as shown in Fig. S4. For $\eta P_{\mathrm{in}}/P_0$ greater than the threshold of 1.54, there is a range of δ over which the solution where $P_{\mathrm{CW}} = P_{\mathrm{CCW}}$ is unstable; instead the symmetry breaks and the coupled powers split, accompanied by a splitting between the resonance frequencies in the two directions.

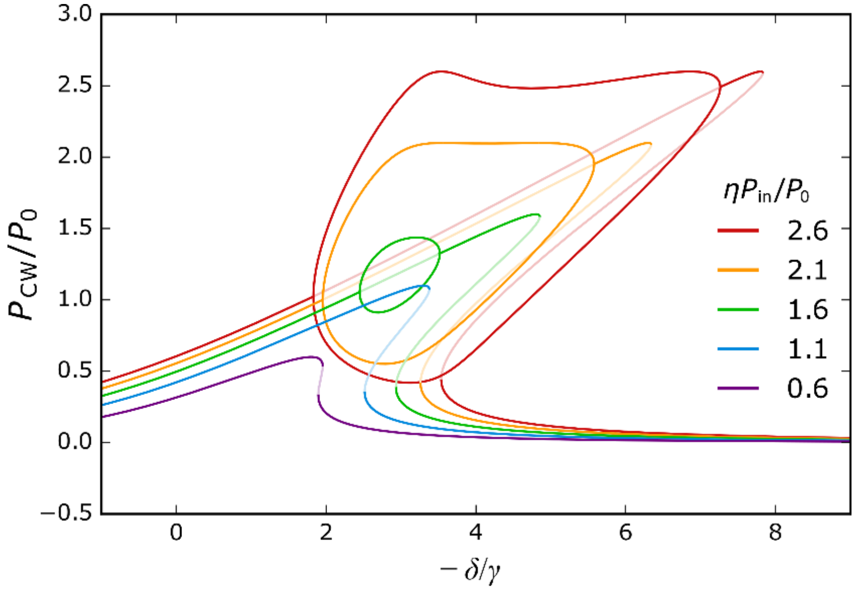

Fig. S4 | Solutions to equations (1, 2) under the condition of equal pump powers $\boldsymbol{P_{\mathrm{in,CW}} = P_{\mathrm{in,CCW}} \equiv P_{\mathrm{in}}}$. The graph shows the power $\boldsymbol{P_{\mathrm{CW}}}$ coupled in the clockwise direction vs. the detuning $\boldsymbol{\delta}$ of the pump light from the resonance without Kerr-shift for different values of $\boldsymbol{P_{\mathrm{in}}}$. Feint lines correspond to unstable solutions. For $\boldsymbol{\eta P_{\mathrm{in}}/P_0 > 8/(3\sqrt{3}) \approx 1.54}$, there is a range of $\boldsymbol{\delta}$ for which two stable solutions exist; when $\boldsymbol{P_{\mathrm{CW}}}$ adopts one, its counterclockwise counterpart $\boldsymbol{P_{\mathrm{CCW}}}$ adopts the other.

Producing the hysteresis graph

In Fig. 2c we display the power coupled into the resonator vs. the pump power ratio, taken while holding the laser frequency constant and modulating one of the pump powers

up and down using a Mach-Zehnder EOM. To obtain the coupled power, we measured the power transmitted by the tapered fibre vs. the voltage applied to the EOM both with and without the resonator, and then subtracted the two traces. The measurements of the output power of the EOM taken via a 1% coupler were used to represent the horizontal axis as a pump power ratio.

Mach-Zehnder interferometer for Fig. 4b

When using coherent counter-propagating light derived from the same source in fibre optics it is important to consider interference effects due to unwanted reflections off connectors and optical components. Since the light is travelling in both directions, any reflected light interferes with the light travelling in the opposite direction. The interference changes from constructive to destructive with the laser frequency f since the relative phase ϕ of the two waves changes as:

$$\phi = 2\pi f \frac{(L_1 - L_2)}{c} n \tag{S4}$$

where L_1 and L_2 are the two path lengths from the point where the light splits (in the 3dB splitter right after the amplifier) to where it recombines, i.e. the reflection point, and n is the refractive index of the fibre. Even a tiny reflection will cause a relatively large power fluctuation, e.g. a -40 dB reflection will cause a ±2% fluctuation as the interference phase changes.

It is possible to reduce this effect by choosing high-return-loss components and ensuring that any fibre-to-fibre connection is clean. The residual power oscillation can be dealt with by setting the interference phase to be almost constant by balancing the two path lengths to the reflection point, although this does not work if there are multiple reflection points. Optical isolators can help to remove reflections from parts of the setup which are far from the tapered fibre, though they may introduce reflections of their own.

In our measurements we took advantage of this effect to create the ±3% pump power imbalance oscillations for Fig. 4b. Furthermore, tuning the path length difference allowed us to choose the power oscillation period with respect to the laser frequency change.

Comb generation and other non-linear effects

The threshold power for symmetry breaking ($\eta P_{\text{in, CW}}/P_0 > 8/(3\sqrt{3}) \approx 1.54$) is identical to the minimum possible threshold for sideband generation by four-wave mixing[2]. However, the actual threshold for sideband generation is only this low if the Kerr shift of the sidebands due to cross-phase modulation from the pump exactly cancels out the resonator dispersion, which is rarely the case. As a result, the nonlinearity-induced symmetry breaking between counter-propagating light usually appears before four-wave mixing sidebands are generated. To avoid this unwanted effect we simply chose a mode family that does not exhibit any other nonlinearities, which we confirm with an OSA. In addition, other nonlinearities are very obvious in the transmitted power through the tapered fibre since they change the amount of light in the pump mode. We could also observe competing nonlinear optical effects such as Raman scattering and Brillouin scattering, which can lead to more complicated dynamics of the optical system. However, Brillouin scattering has a quite narrow gain bandwidth with ~10 GHz shift from the pump laser, which is not resonant in our microresonator. Raman scattering can be quenched using the right coupling regime[3].

Derivation of equations 2 and 3

In high-Q dielectric microresonators, it is possible to reach sufficient circulating intensities to see the effects of the Kerr, or χ^3, nonlinearity. The refractive index n of a mode in a cicular resonator increases linearly with the circulating power P_{circ} in that mode:

$$\frac{\partial n}{\partial P_{\text{circ}}} = \frac{n_2}{A_{\text{eff}}} \qquad (S5)$$

Where n_2 is the nonlinear refractive index of the material and A_{eff} is the effective made cross-sectional area. This causes a decrease in the resonance frequency known as self-phase modulation:

$$\frac{\partial \omega_{\text{res}}}{\partial P_{\text{circ}}} = -\frac{\omega_{\text{res}}\, n_2}{n_0\, A_{\text{eff}}} \qquad (S6)$$

Where n_0 is the resonator's linear refractive index i.e. that without any Kerr shift. It is well known that the Kerr shift induced on counterpropagating light of the same polarisation (i.e. cross-phase modulation) is twice as strong as the self-phase modulation. The resonance frequency of a clockwise (CW)-propagating mode thus depends on its own circulating power and that of the corresponding counterclockwise (CCW)-propagating mode as:

$$\frac{\partial \omega_{\text{res, CW}}}{\partial P_{\text{circ, CW}}} = -\frac{\omega_{\text{res}}\, n_2}{n_0\, A_{\text{eff}}} \qquad \text{and} \qquad \frac{\partial \omega_{\text{res, CW}}}{\partial P_{\text{circ, CCW}}} = -2\frac{\omega_{\text{res}}\, n_2}{n_0\, A_{\text{eff}}} \tag{S7}$$

and vice versa for the CCW mode. These shifts modify the detunings in the usual Lorentzian expressions for the circulating powers:

$$P_{\text{circ, CW}} = \frac{2\kappa\, \Delta f_{\text{FSR}}\, P_{\text{in, CW}}}{\gamma^2 + \left(\delta + \frac{\omega_{\text{res}}\, n_2}{n_0\, A_{\text{eff}}}\left(P_{\text{circ, CW}} + 2P_{\text{circ, CCW}}\right)\right)^2} \tag{S8}$$

$$P_{\text{circ, CCW}} = \frac{2\kappa\, \Delta f_{\text{FSR}}\, P_{\text{in, CCW}}}{\gamma^2 + \left(\delta + \frac{\omega_{\text{res}}\, n_2}{n_0\, A_{\text{eff}}}\left(P_{\text{circ, CCW}} + 2P_{\text{circ, CW}}\right)\right)^2} \tag{S9}$$

Here $\gamma = \gamma_0 + \kappa$ is the loaded half-linewidth of the resonance wherein γ_0 and κ are the intrinsic and coupling half-linewidths, $P_{\text{in, CW}}$ and $P_{\text{in, CCW}}$ are the incident powers, Δf_{FSR} is the free spectral range of the mode family and δ is the detuning of the pump laser from the resonance without Kerr shift. These expressions can be simplified by making use of the coupled powers $P_{\text{CW}} = 2\pi\, P_{\text{circ, CW}}/F_0$ and $P_{\text{CCW}} = 2\pi\, P_{\text{circ, CCW}}/F_0$ where $F_0 = \pi\, \Delta f_{\text{FSR}}/\gamma_0$ is the intrinsic finesse to give equations (2) and (3), using the in-coupling efficiency $\eta = 4\kappa\gamma_0/\gamma^2$ and defining the characteristic power

$$P_0 = \frac{\pi\, n_0\, A_{\text{eff}}}{Q\, F_0\, n_2} \tag{S10}$$

at which Kerr nonlinear effects occur, wherein $Q = \omega_{\text{res}}/(2\gamma)$ is the loaded quality factor.

References:

1. Del'Haye, P., Diddams, S. A. & Papp, S. B. Laser-machined ultra-high-Q microrod resonators for nonlinear optics. *Applied Physics Letters* **102**, 221119 (2013).
2. Matsko, A. B., Savchenkov, A. A., Strekalov, D., Ilchenko, V. S. & Maleki, L. Optical hyperparametric oscillations in a whispering-gallery-mode resonator: Threshold and phase diffusion. *Physical Review A* **71**, 033804 (2005).
3. Kippenberg, T. J., Spillane, S. M. & Vahala, K. J. Kerr-nonlinearity optical parametric oscillation in an ultrahigh-Q toroid microcavity. *Physical Review Letters* **93**, 083904 (2004).
